# Artificial Intelligence Technologies in Nursing Clinical Decision‐Making: An Umbrella Review

**DOI:** 10.1111/jan.70579

**Published:** 2026-03-17

**Authors:** Robyn Cant, Colleen Ryan, Ritesh Chugh

**Affiliations:** ^1^ Health Innovation and Transformation Centre Federation University Australia Berwick Victoria Australia; ^2^ School of Nursing, Midwifery and Social Sciences, CREATE Research Centre Central Queensland University Brisbane Queensland Australia; ^3^ School of Engineering and Technology, CML‐NET & CREATE Research Centres Central Queensland University Melbourne Victoria Australia

**Keywords:** artificial intelligence, clinical decision‐making, data analytics, machine learning, nursing

## Abstract

**Aim:**

To describe contemporary peer‐reviewed literature on artificial intelligence in nurses' clinical decision‐making.

**Methods:**

An umbrella review of literature reviews.

**Data Sources:**

Four major databases were searched for reviews published between 2019 and 2024.

**Results:**

Sixteen literature reviews reported on 965 nursing artificial intelligence primary studies. The studies focused on technology development and emerging performance evaluations, whilst real‐world testing or implementation in nursing clinical settings was rare. Rigorous comparative analyses were lacking. While artificial intelligence demonstrates promise in decision‐making, challenges such as a lack of controlled studies, algorithmic bias, limited reproducibility and insufficient clinical trials hinder its practical impact. Ethical concerns, transparency and patient data privacy issues pose barriers to AI integration in nursing practice. Ethical and legal guidelines for patient privacy are needed and should be taught along with AI literacy training for nurses.

**Conclusions:**

Artificial intelligence has the potential to enhance clinical nursing decision‐making, although evidence is limited by too few examples of nurse participation during development. Underutilisation in administrative nursing functions hinders implementation. Nurses should assume a central role in the design and development of AI applications to ensure that these technologies address the realities of nursing practice. With such improvements, artificial intelligence can transform nursing practice, improve nurses' clinical decision‐making and ultimately enhance consumer healthcare outcomes.

**Patient or Public Involvement:**

No Patient or Public Involvement.

**Reporting Method:**

While there is no reporting checklist for umbrella reviews, the PRISMA guide for systematic reviews was followed.

## Implications for the Profession and/or Patient Care

- What problem did the study address?
  ○ Artificial intelligence applications have long been utilized in medicine for clinical decision‐making; however, a lack of uptake in nursing decision‐making should be remedied.
- What were the main findings?
  ○ AI can transform nursing practice by improving nurses' clinical decision‐making; however, stronger evidence about applications and AI literacy training is necessary.
- Where and on whom will the research have an impact?
  ○ The findings will impact curriculum designers, clinical facilitators and professional nurses who may translate findings into practice improvements.

## 1 Introduction

Artificial intelligence (AI) has shown promise for use in healthcare and nursing domains in the last decade, especially concerning patient care and educational processes for nursing staff (Lifshits and Rosenberg 2024). While AI is often defined as ‘a system's ability to correctly interpret external data, to learn from such data and to use those learnings to achieve specific goals and tasks through flexible adaptation’ (Kaplan and Haenlein 2019, 17), this definition reflects an aspirational view rather than a definite outcome. In practice, AI algorithms are prone to errors, bias and misinterpretation of data—factors that pose risks when applied in clinical environments (Yelne et al. 2023). Computer‐based statistical algorithms have nonetheless demonstrated the ability to recognise patterns and extract insights from large datasets (Jothi and Husain 2015) and are trained to learn from available data and to use that knowledge to perform analyses without explicit programming. AI has facilitated the development of innovative tools for practical applications (Ahmed et al. 2023).

Nurses' decision‐making is a complex process that integrates clinical experience with critical thinking (Nibbelink and Brewer 2018). This requires nurses to make rapid decisions by evaluating subjective and objective evidence, determining its relevance as essential, critical or irrelevant before taking action (Johansen and O'Brien 2016). AI can assist nurses by promoting the decision‐making process and identifying the best action to take (Ng et al. 2022). These authors reported that various machine learning and classification techniques were used in predictive analyses to improve nurses' preparedness and management of patients' conditions. Thus, AI represents a new frontier that can assist nurses in decision‐making.

### 1.1 AI Technologies

AI is a field of computer science concerned with developing systems capable of performing tasks that typically require human intelligence, such as reasoning, learning, problem‐solving and understanding language. AI encompasses various subfields and techniques, including machine learning (ML), deep learning and natural language processing (NLP), each contributing to the design of intelligent systems with diverse capabilities (Mahesh 2020).

Machine learning, a core component of AI, enables systems to learn from data by identifying trends and patterns, enabling improved performance over time without being explicitly programmed for every scenario (Alpaydin 2020). ML techniques are typically classified into three main paradigms: supervised learning, unsupervised learning and reinforcement learning. In supervised learning, models are trained on labelled datasets to perform predictive tasks such as classification or regression; commonly used algorithms include support vector machines, decision trees and random forests. Unsupervised learning focuses on uncovering hidden patterns or structures in unlabelled data, employing techniques such as clustering (e.g., k‐means) or dimensionality reduction (e.g., principal component analysis). Reinforcement learning involves agents making sequential decisions in dynamic environments by maximising cumulative rewards through interactions (Padakandla 2021).

In addition to these paradigms, machine learning also includes algorithmic families that span across categories. For example, ensemble learning combines multiple base models to enhance prediction accuracy and robustness. Random forest algorithm is a widely used example, which is based on aggregating the outcomes of multiple decision trees built on different subsets of data to improve generalisation and reduce overfitting (Schonlau and Zou 2020; Sun et al. 2024). Another major family is deep learning, a subset of ML involving multilayered neural networks that process data through hierarchical layers. These networks are inspired by the structure and function of the human brain and are particularly effective in handling large‐scale, high‐dimensional data (Bhuyan et al. 2025; Yousef and Allmer 2023).

Natural language processing (NLP) is a field of AI concerned with enabling computers to interpret, analyse, generate and respond to human language. It is not a machine learning paradigm in itself, but rather an application domain that employs a variety of ML methods, including both supervised and unsupervised approaches. NLP supports applications such as speech recognition, automated summarisation, language translation and chat‐based interfaces (Khurana et al. 2023).

While Generative AI may be more familiar to some readers, it differs from many traditional AI applications in that it is designed to produce new content such as images, text or data, rather than identifying patterns or making predictions. It uses advanced ML models, often large‐scale deep learning architectures, to generate outputs that mimic the style or characteristics of the training data. This distinguishes generative AI from conventional predictive models and has implications for creative and knowledge‐based domains (Helen and Suresh 2024; Park et al. 2024).

The growing integration of AI technologies across industries highlights their potential to enhance efficiency, support decision‐making and shape the future of decision‐making and problem‐solving (Kaggwa et al. 2024; Montejo et al. 2024). In healthcare, and in nursing specifically, the use of AI is growing. In a bibliometric analysis of AI applications in nursing research, Douthit et al. (2022) found that supervised learning methods, particularly ensemble techniques such as random forests, were the most commonly applied.

AI has long played a role in medical domains, particularly in clinical decision support systems that assist with diagnosis prediction, risk stratification, screening for preventable diseases and avoidance of adverse events (Garg et al. 2005). However, scholars have noted a lack of nursing‐specific involvement in the design, implementation and evaluation of AI tools. Fernandes et al. (2020) advocate for greater nurse participation in AI research, especially in high‐pressure environments such as emergency care and triage. This calls attention to the urgent need for nurses to be educated, trained and actively involved in understanding and applying AI technologies in clinical settings (Montejo et al. 2024; Park et al. 2024).

Despite a rapid increase in AI‐focused nursing publications over the past 5 years (as indexed by the National Library of Medicine), there remains limited insight into the profession's broader adoption of AI, particularly in the context of clinical decision‐making. This gap suggests that more targeted review and synthesis of emerging evidence are needed to inform the development of nursing‐specific AI practices, policies and educational resources (Yim et al. 2024). It is timely to employ review methods to advance the field through critical abstraction and synthesis of the growing body of evidence.

### 1.2 The Review

Despite an escalation in AI studies in nursing in recent years, there is a lack of comprehensive understanding of how AI is utilised in nursing practice, and the uptake of these technologies has been slow (Shang 2021). We present a review of peer‐reviewed reviews of literature, summarising the work of authors who have previously conducted literature reviews on the topic. The findings are organised under an overarching theme or ‘umbrella’ (Belbasis et al. 2022) labelled with the generic term ‘AI in nursing’.

This umbrella review aims to examine contemporary reviews of the literature on artificial intelligence applications for nurses' clinical decision‐making and to explore how the results relate to the quality of patient care.

## 2 Methods

A review of reviews, often termed an umbrella review, compiles evidence from a number of existing reviews to facilitate comparison between the studies. This review was informed by an umbrella review method described by Cant et al. (2022) to provide an analysis of the research evidence of AI integration with nurses' clinical decision‐making. Umbrella reviews are valuable when numerous empirical studies on a specific topic have been published because they offer a comprehensive overview, documenting the study quality and highlighting areas of uncertainty (Belbasis et al. 2022).

The review was conducted between September and November 2024. The PICO mnemonic (Population, Intervention, Comparator, Outcome) was used to design review questions to extract the key findings. The research questions (RQ) addressed are:

1. What is the evidence for the validity and integrity of studies of AI in nursing?
2. In what areas of nursing does AI research report an actual impact or potential benefit for nurses' clinical decision‐making?
3. How effectively do AI applications transfer into clinical nursing practice?

### 2.1 Inclusion Criteria

The population for this review was professional nurses, with studies of all levels of qualified nurses eligible for inclusion. English language publications within the last 5 years (2019–2024) were selected to ensure a review of contemporary research and recent application of artificial intelligence technologies. Table 1 describes the inclusion and exclusion criteria.

**TABLE 1:** Inclusion and exclusion criteria.

Inclusion criteria	Exclusion criteria
Peer reviewed Reviews of literature Professional nurse Registered nurse Artificial intelligence Machine learning Data mining Publications in English Publications 2019–2024	Medicine Professions allied to medicine Clinical practice standards Nurse education Students Primary studies Grey literature Conference papers

### 2.2 Literature Search Strategy

Structured searches of the literature were conducted across four major databases: PubMed, Scopus, Web of Science and The Cochrane Database of Systematic Reviews. The search was limited to English‐language publications over 5 years, from 2019 to 2024. The primary search terms, informed by the PICO framework, were ‘nursing’ (population), ‘artificial intelligence’ (intervention) and clinical decision‐making (outcome). These terms were combined with other keywords in different search phrases to suit each database and were applied with Boolean operators. The search results indicated that most studies focused on medical management or disease classification, which were unrelated to nursing clinical decision‐making and were therefore excluded. To ensure the identification of recent studies, an additional search was conducted using Google Scholar, and a manual review of reference lists from the included studies was performed. The first two authors independently performed searches.

A total of 396 literature reviews were identified across library databases: 317 from PubMed, 100 from Web of Science, and 79 from Scopus, with none from the Cochrane Database of Systematic Reviews. In addition, 15 reviews were identified through Google Scholar, while no relevant studies were found in the reference lists of the included reviews.

### 2.3 Data Extraction and Synthesis

**FIGURE 1:**
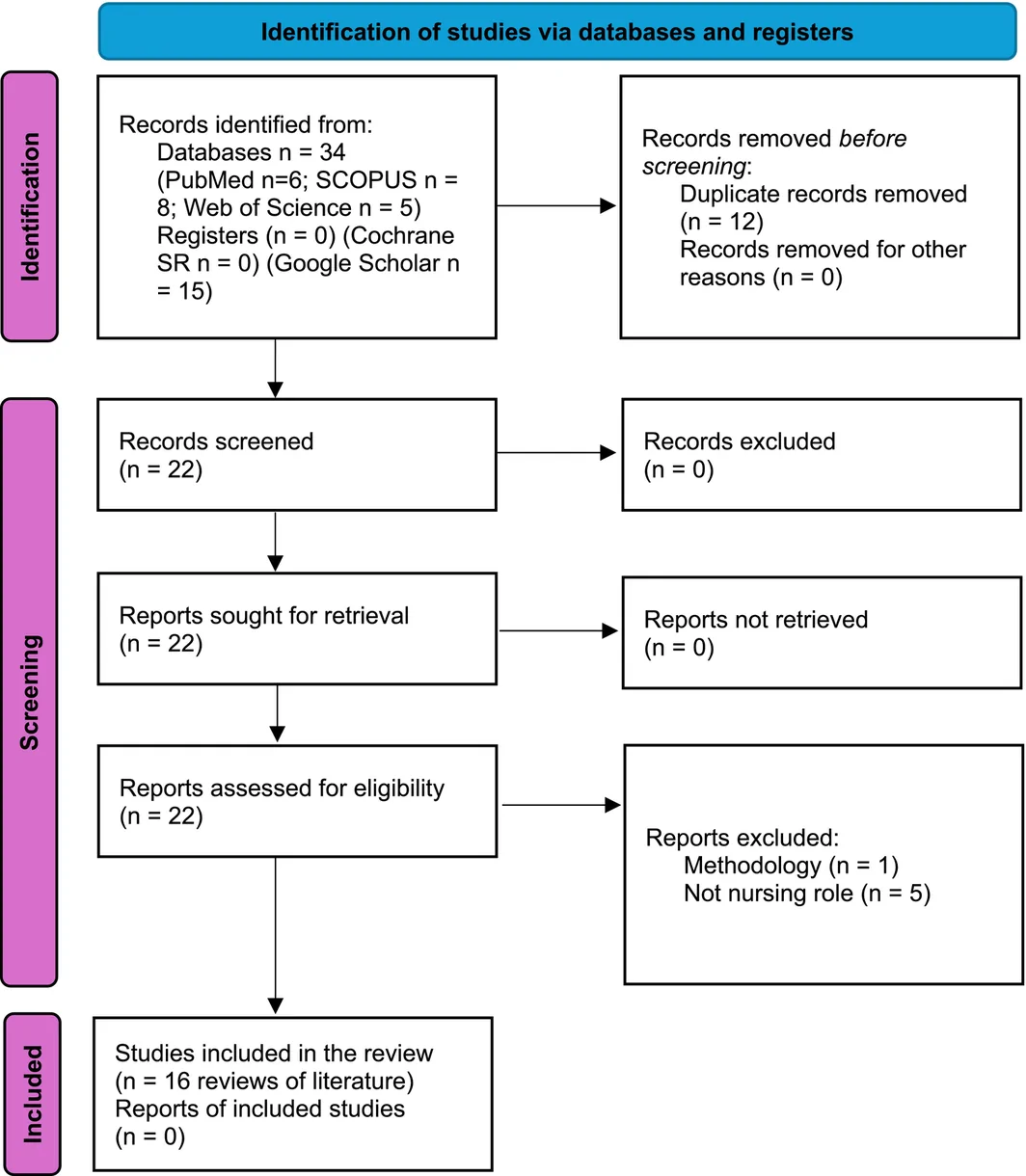
PRISMA flow chart.

The list of review studies was screened by applying the inclusion and exclusion criteria, focusing on topics closely aligned with nursing roles. Figure 1 presents the PRISMA chart outlining the study identification and selection process.

The full‐text content of each potential article was assessed for relevance and alignment with the review's scope. The three authors held online discussions to reach a consensus on the final selection of included reviews.

The final sample was 16 literature reviews of AI studies in nursing that were published between 2019 and 2024. The characteristics of each article were systematically tabulated across six elements to allow comparison: main author/year of publication/article title; design/sample/designs in primary studies; aim; result; and conclusions. A formal quality assessment was not conducted as there is no recognized standard for umbrella reviews. The authors collaborated to extract and cluster the review results and the conclusions to facilitate a summary and synthesis of findings to answer the research questions, as follows.

## 3 Results

All 16 literature reviews focused on the nursing profession and the integration or potential application of AI research in nurses' clinical decision‐making.

### 3.1 Characteristics of Included Reviews

Of the 16 reviews, eight (50%) were systematic reviews, primarily comprising cohort studies (Chua et al. 2021; Hossain et al. 2023; Kikuchi 2020; Martinez‐Ortigosa et al. 2023; O'Connor et al. 2023; Ruksakulpiwat et al. 2024; Sánchez‐Salmerón et al. 2022; Xie et al. 2022). Four were scoping reviews (Buchanan et al. 2020; Choi et al. 2024; Ng et al. 2022; von Gerich et al. 2022), one was a rapid review (Seibert et al. 2021), and three did not specify their review methods (Jeong 2020, Mohanasundari et al. 2023; Yelne et al. 2023).

The level of evidence presented across the reviews varied, but no study was excluded due to lower levels of evidence because each contributed relevant findings. The total number of articles reviewed across all studies was 965, with individual reviews ranging from 11 to 292 studies. Three reviews did not report the number of included studies. Table 2 summarises characteristics of the reviews.

**TABLE 2:** Characteristics of reviews of AI research in nursing.

Author (year); country of origin	Design/sample	Aim	Result	Conclusion
Buchanan et al. (2020); Canada	Scoping review; *N* = 131 articles; all designs in the types of studies.	To summarise emerging/predictive trends in AI‐powered health technology and implications for nursing administration, clinical practice, policy and research.	AI is used to influence nursing roles/workload (documentation), workflows and the nurse–patient relationship. Robots are not viewed as nurses' clinical decision‐making replacement tools. Clinical nurses used AI reducing time spent on clinical decision‐making in nursing diagnoses.	Nurses need to drive the use of AI in nursing practice and education. More use of AI and ML for clinical decision‐making may reduce time spent on tasks such as documentation, allowing for more time spent with patients.
Choi et al. (2024); USA	Scoping review; *N* = 26 studies; Some were single studies, no controlled studies included. Some studies did not report the instruments used.	To report the most effective machine learning algorithms and in what areas of nursing ML is used.	ML is used in decision‐making around nurses' workload and mental health and wellbeing in areas like the emergency department where there are large data sets. Clinical decision‐making for preventing falls, infection and pressure injuries featured in 8 studies only. Random forest was the most common algorithm.	ML is used successfully to enhance treatment decisions and improve patient care. Nursing needs to consider more applications of the technology to improve clinical decision‐making.
Chua et al. (2021); Australia	Systematic review; Six retrospective single centre observational studies were included.	Can big data predict the incidence of delirium in the inpatient setting.	The best performing model for delirium prediction was random forest. There was much heterogeneity in delirium definitions and screening. Studies were of low quality with outcomes, sample size and analysis methods unclear.	Data mining techniques can be applied to large data sets to accurately predict delirium in inpatient settings. Despite heterogeneity and low quality results indicate ML techniques demonstrate consistent and strong predictive capabilities in identifying and understanding delirium.
Hossain et al. (2023); Bangladesh	Systematic review; [127 papers regarding EHR most in the core group that listed data were experimental (𝑛 = 24), cohort (𝑛 = 9), case study (𝑛 = 1)].	A systematic review guided by PRISMA reviewed 7 categories of AI articles: (1) medical note classification, (2) clinical entity recognition, (3) text summarisation, (4) deep learning (DL) and transfer learning architecture, (5) information extraction, (6) medical language translation and (7) other NLP applications.	Electronic health records were the most commonly used data type & datasets were mainly unstructured. Various ML and deep learning methods were used.	Deep learning algorithms have achieved great success in natural language processing, although application in the biological realm remains difficult. Transformer‐based models for free text analysis are yet to be used extensively, as conventional methods are currently preferred.
Jeong (2020); Korea	Review methods not described including the number of articles. Reference list has descriptive study citations.	To review the concepts of AI, ML and nursing research; the necessity of education on AI in nursing schools; and areas of nursing care where AI is useful.	AI using nursing clinical notes is used in many situations. E.g., prenatal nursing interventions according to pregnant women's nursing records, prediction of risk of delivery, prediction of postpartum depression, risk of breast cancer before/after menopause, the needs for nursing care among elderly women with physical disabilities, adverse medication events and trends in women's health nursing research and practice.	AI, including ML and deep learning, are inevitable trends in nursing research, education and practice. Nurses need to understand the principles of AI and apply AI in a broad number of settings.
Kikuchi (2020); Japan/USA	Systematic review; *N* = 17 articles, mostly descriptive studies using large datasets, including tool development evaluations (No SR).	To synthesise nursing studies leveraging artificial intelligence technology and to offer suggestions for future studies.	AI technology performs well in predicting outcomes AI technology is used for nursing clinical and management decision‐making in falls prediction, surgery‐related injuries, patient survival. Management areas were staff retention, safety, mental wellbeing, education outcomes, predicting patient admissions, bed allocations and communication risks.	AI has the potential to help develop nursing science and practice. Need interdisciplinary approaches, including multicentre collaborative research. Nurses should use appropriate wording n the (electronic) medical record to facilitate more research based on natural language processing, to identify hidden patient data to assist nurses' clinical decision making.
Martinez‐Ortigosa et al. (2023); Spain	Systematic review; *N* = 21 articles, including quantitative and qualitative designs, mostly cross‐sectional studies.	To synthesise the available evidence on the applicability of artificial intelligence in nursing care.	AI applications comprised (i) advances in early disease detection and clinical decision‐making; (ii) AI intelligence‐based support systems in nursing for patient monitoring and workflow optimization; and (iii) AI insights for nurse training and education.	AI‐based systems demonstrated increased autonomy of patients and professionals in care processes such as wound care through guided instructions, improved workflows and efficiency in terms of time, materials and human resources.
Mohanasundari et al. (2023); India	Review method is not recorded, mostly descriptive studies in the reference list, no controlled studies.	To review the literature on AI and describe the necessity for careful integration in healthcare, acknowledging its advantages while mitigating drawbacks.	The role of AI should be to complement, not replace, the unique skills and empathetic aspects of nursing care. Addressing concerns related to bias, transparency, data privacy and professional training is essential to maximise the benefits of AI in healthcare.	AI has a transformative potential in healthcare. Improving efficiencies and patient care through administrative tasks, early disease detection, personalised treatment plans, predictive analytics and surgical interventions, Implementation challenges include the potential for errors and biases in algorithms, lack of transparency in decision‐making, patient privacy/ethical issues, dependency leading to skill decline, high costs and resistance to change within the healthcare ecosystem.
Ng et al. (2022); Singapore	Scoping review; *N* = 37 articles: mostly uncontrolled developmental studies, no controlled studies or SR.	To present an overview of how artificial intelligence has been used to improve clinical nursing care.	Six uses were identified—documentation, formulating nursing diagnoses, formulating nursing care plans, patient monitoring, patient care prediction (e.g., falls prediction [most common] and wound management). Various machine learning techniques and classification were used for predictive analyses and to improve nurses' preparedness and management of patients.	AI has the potential to improve the quality of nursing care. Next are the need for randomised controlled trials in healthcare settings to enhance the rigour of evidence.
O'Connor et al. (2023); UK/HongKong/Switzerland/Ireland	Systematic review; *N* = 140 articles, mainly quantitative cohort or cross‐sectional designs, many did not report data details, data sets were poor quality and could introduce bias.	To synthesise literature on AI in nursing and midwifery.	AI was mainly applied in clinical practice to direct patient care (82.14%), fewer studies focused on administration, management or education. Few studies involved nurses and midwives.	Most studies trained/tested AI technologies. The benefits were mostly potential, with only 7% having real‐world testing. A risk of bias and limitations were noted. There is a need to develop nurses and midwives' expertise in this rapidly growing area.
Ruksakulpiwat et al. (2024); Thailand/USA	Systematic review; *N* = 11 articles, retrospective cohort studies or tool development (no controlled studies)	To synthesise the available evidence to comprehensively understand the application of AI in nursing care.	Six themes describing AI in nursing for decision making (1) risk identification including disease (osteoporosis/depression) prevention screening, (2) health assessment and (3) patient classification, including assistance with clinical judgement on patient deterioration/severity of illness, (4) research, (5) improved care delivery and medical records and (6) developing nursing care plans.	Findings consolidate the current knowledge base and highlight how AI is shaping and improving nursing care practices. Nursing involvement in developing and implementing AI models is lacking. Nurses' education on AI is lacking. Increased collaboration on planning committees and in AI focused research is needed.
Sánchez‐Salmerón et al. (2022); Spain	Systematic review; *N* = 11 articles; all retrospective studies using medical records.	To analyse the effectiveness of ML systems in triage for making predictions at the emergency department in comparison with other triage scales/scores.	Random forest was best predictor model; Have potential for predicting mortality and in deciding if patients need general admission or critical care interventions.	Predictive ML models showed better results when using medical records combined with nurses' clinical notes.
Seibert et al. (2021); Germany/Denmark	Rapid review; *N* = 292 articles, 77% were level VI (evidence from a single descriptive or qualitative study) and 6 level III controlled non‐randomised studies.	To synthesise literature on application scenarios for AI in nursing care settings and highlight aspects in ethical, legal and social discourses.	AI assisted decision‐making with falls prediction/prevention, pressure ulcers and coordinating care needs assessments. Most studies used machine learning algorithms. Contexts were image and signal processing with tracking, monitoring or classification of activity and health, followed by care coordination and communication, as well as falls detection.	AI has potential to reduce costs, save time, reduce staff workload and improve quality and safety of health care. More dialogue and investigation on the ethical and legal implications of AI is necessary.
von Gerich et al. (2022); Finland, USA, UK	Scoping review; *N* = 93 articles, Designs were generally descriptive, 4.3% of studies included RCT. at Level IV evidence; many lacked study design or aim.	To synthesise currently available state‐of‐the‐artstate‐of‐the‐art research in AI applied in nursing practice.	Most studies explored technology development (*n* = 55, 59.1%) and formation (testing) (*n* = 28, 30.1%) phases. Only one‐third involved nurses; these were mostly ward nurses with some advanced practice nurses. Technologies were designed for disease and risk prediction, tracking and monitoring patients, care plans and clinical notes. The only area of evidence for AI in operation was risk prediction.	Levels of evidence by designs was low. Reporting lacked detail of aims, setting and methods. Contemporary research on applications of AI‐based technologies in nursing mainly cover early stages of technology development, with scarce evidence of the impact and implementation into practice.
Xie et al. (2022); China	Systematic review and meta‐analysis; *N* = 22 studies, *n* = 17 cohort studies plus four case–control. Only four were complete and were statistically meta‐analysed.	To critically appraise and quantify the performance studies by employing ML to predict delirium.	All models showed moderate to excellent performance in detecting delirium, but lacked external validation. In meta‐analysis, the receiver operating characteristic curve for predicting delirium was 0.89, sensitivity 0.85 (95% confidence interval 0.84–0.85) and specificity 0.80 (95% confidence interval 0.81–0.80).	All four ML models meta‐analysed showed excellent performance in predicting delirium. Most studies had a high risk of bias, especially owing to missing data. As most were observational, it was difficult to compare studies. More rigorous evaluations of models are needed.
Yelne et al. (2023); India	Review method not described, addresses nursing and healthcare, 69 citations, often descriptive studies, includes SRs.	To critically examine the multifaceted impact of AI on nursing science and healthcare.	AI's capacity to improve patient outcomes, enhance efficiency and propel medical research is undeniable. However, the ethical considerations surrounding transparency, fairness and accountability remain as concerns.	AI complements human healthcare providers, as the human touch and empathy remain irreplaceable. Continued research, interdisciplinary collaboration, ethical guidelines and protection of patient rights require attention to realise AI's full potential in healthcare.

*Note:* The reviews prioritise general nursing practice. The following specialty studies were not included in the reviewed literature: medical decision‐making; aged care, cancer care, cardiovascular management, dentistry, education, infectious diseases, IVF, paediatrics, palliative care, pathology, radiology, rehabilitation and surgery.

Abbreviations: AI, artificial intelligence; ML, machine learning.

### 3.2 Evidence for Validity and Integrity of Studies of AI in Nursing (RQ1)

In response to RQ1: What is the evidence for the validity and integrity of studies of AI in nursing?, the primary research included in each review comprised a variety of research designs. As reported in Table 2, the majority were descriptive (cohort) studies, with few involving experimental or controlled designs that can provide higher‐level evidence. To highlight this issue, Table 3 presents a clinical guide to denote the hierarchy of research evidence.

**TABLE 3:** The hierarchy of research evidence.

Level	Descriptor
I	Evidence obtained from a systematic review of all relevant randomised control trials.
II	Evidence obtained from at least one well‐designed randomised control trial.
III	Evidence obtained from well‐designed controlled trials without randomisation.
IV	Evidence obtained from well‐designed cohort studies, case–control studies, interrupted time series with a control group, historically controlled studies, interrupted time series without a control group or with case‐series.
V	Evidence obtained from systematic reviews of descriptive and qualitative studies.
VI	Evidence obtained from single descriptive and qualitative studies.
VII	Expert opinion from clinicians, authorities and/or reports of expert committees or based on physiology.
	References: evidence based on these reports: National Health and Medical Research Council (2009). NHMRC levels of evidence and grades for recommendations for developers of guidelines (2009). Australian Government: NHMRC. http://www.nhmrc.gov.au/_files_nhmrc/file/guidelines/evidence_statement_form.pdf. Melynyk, B. and Fineout‐Overholt, E. (2011). Evidence‐Based Practice in Nursing & Healthcare: A Guide to Best Practice (2nd ed.). Philadelphia: Wolters Kluwer, Lippincott Williams & Wilkins. OCEBM Levels of Evidence Working Group Oxford (2011). The Oxford 2011 Levels of Evidence. Oxford Centre for Evidence‐Based Medicine. http://www.cebm.net/index.aspx?o=1025.

*Source:* The Royal Children's Hospital Melbourne.

Several emerging concepts are relevant to the review question: What is the evidence for the validity and integrity of studies of AI in nursing? First, the pattern of primary citations in AI nursing research suggests that the overall evidence base consists of studies that lie within the lower range of the evidence hierarchy, Level III or Level IV. Overall, empirical studies incorporating a comparison group were rare. As shown in Table 3, randomised or non‐randomised designs with a control group provide higher evidence (Level 11).

Several review authors highlighted low levels of evidence in the primary studies. In particular, von Gerich et al. (2022) noted low levels of research evidence in primary study designs. Chua et al. (2021) found that six cohort studies of delirium identification tools in their review were of low to moderate quality. Xie et al. (2022) noted wide variation in reporting methodological elements among the mostly cohort‐based studies to detect delirium, suggesting that comparisons between studies were therefore made difficult.

In their review, von Gerich et al. (2022) noted that over half the studies (55/93) addressed the development of technology, and only around one‐third (28/93) tested AI applications, with few studies involving nurses. Overall, most validation efforts were centred around evaluating performance measures, typically through user processing or researcher evaluation of performance based on confidence in the intended purpose. Although patient outcomes in the above‐named review were the focus for 60% of studies, results identified a lack of testing applications in real‐world settings with nurses. Table 4 examines this theme by summarising the content of the reviews regarding how they approached reporting analysis and evaluation. We categorised the degree of maturation in reported elements of AI applications: either development, testing, or usage, or several of these. Several reviews summarised only potential applications.

**TABLE 4:** The focus and maturation of AI review technologies research in nursing.

Review		Methods			
Study	Topic	Application development	Application testing	Application usage^a^	Proposed use
Buchanan et al. (2020)	Domains of nursing				✔✔✔
Choi et al. (2024)	Effective algorithms		✔✔✔	✔✔✔	✔✔✔
Chua et al. (2021)	Delirium prediction		✔✔✔	✔✔✔	
Hossain et al. (2023)	Electronic Health records and algorithms		✔✔✔	✔✔	
Jeong (2020)	AI/women's health nursing				✔✔✔
Kikuchi (2020)	AI in nursing studies	✔✔	✔✔	✔✔	
Martinez‐Ortigosa et al. (2023)	Nursing care			✔✔✔	
Mohanasundari et al. (2023)	Future of AI and algorithms			✔✔✔	✔✔✔
Ng et al. (2022)	Nursing care	✔✔✔		✔✔✔✔	
O'Connor et al. (2023)	Nursing care	✔✔✔	✔✔✔	✔	
Ruksakulpiwat et al. (2024)	Nursing care	✔✔			✔✔✔
Sánchez‐Salmerón et al. (2022)	Triage models		✔✔✔		
Seibert et al. (2021)	Nursing care and algorithms			✔✔	✔✔✔
von Gerich et al. (2022)	Nursing care	✔✔✔	✔✔✔		
Xie et al. (2022)	Delirium prediction			✔✔	✔✔✔
Yelne et al. (2023)	Nursing care			✔✔	✔✔✔

*Note:* Some content = ✔; moderate content = ✔✔; large content = ✔✔✔.

^a^ ‘Application usage’ is either actual outcomes reported, or retrospective descriptive inferences.

A summary of AI application evaluation methods was provided by von Gerich et al. (2022). The most cited evaluation methods were performance evaluation measures (75.3%, *n* = 70), user evaluation (19.4%, *n* = 18), comparative evaluation not including performance measures (6.5%, *n* = 6), and quality evaluations (3.2%, *n* = 3).

Only one review examined AI algorithms (Choi et al. 2024), although several reported some algorithm details (Hossain et al. 2023; Mohanasundari et al. 2023; Sánchez‐Salmerón et al. 2022; Seibert et al. 2021). A review by Sánchez‐Salmerón et al. (2022) found that most studies used machine learning algorithms, with random forest identified as the most accurate. Similarly, Choi et al. (2024) reported that random forest was the most frequently used machine learning method. Although deep learning algorithms have been successful in natural language processing, Hossain et al. (2023) reported that they may be difficult to apply in biological studies. Mohanasundari et al. (2023) identified that algorithmic bias could pose a challenge for implementation and be difficult to correct.

The pattern of primary studies focusing on preliminary aspects, such as the development of AI applications, was mirrored in several studies. O'Connor et al. (2023) found that of 140 reviewed studies of AI in nursing care, most involved the development and/or testing of applications, with only 7% reporting actual benefits of AI as a result of intervention studies. Xie et al. (2022), in a review of AI models to detect patient delirium, found that although the instruments were excellent at predicting cases of delirium, there was a high risk of bias and a lack of reproducibility due to the variability in reporting and missing data (which raises concerns about the validity of these studies). Similarly, Ng et al. (2022) found that machine learning techniques and disease classification tools were applicable for predictive analyses and for improving nurses' preparedness and patient management. However, they expressed a need for stronger evidence of the impact of AI research outcomes on practice, particularly through reporting more randomised controlled trials conducted in real‐life healthcare settings.

### 3.3 AI Applications in Nursing Decision‐Making (RQ2)

In response to RQ2: In what areas of nursing does AI research report an actual impact or potential benefit for nurses' clinical decision‐making?, there were wide‐ranging responses in the literature. Table 5 presents a summary list of AI application contexts drawn from the reviewed literature.

**TABLE 5:** Proposed or actual artificial intelligence applications in nursing literature reviews^a^.

Clinical area	Focus of AI algorithm	References
Acute care clinical nursing	Documentation Nursing diagnoses Nursing care plans Patient monitoring Patient care prediction (e.g., fall prediction; wound management)	Ng et al. (2022)
Clinical care	Early disease detection AI support systems for clinical decision‐making Nurse workflow optimisation	Martinez‐Ortigosa et al. (2023)
General health care	Risk identification, including disease prevention screening (e.g., osteoporosis/depression) Health assessment Patient classification, *including clinical judgement on patient deterioration/severity of illness* Improved care delivery and medical records Developing nursing care plans Research	Ruksakulpiwat et al. (2024)
Emergency department triage	Prediction of emergency hospital admission Prediction of ICU admission	Sánchez‐Salmerón et al. (2022)
General nursing care	Tracking, monitoring patients Classification of activity and health care Care coordination Communication Falls detection	Seibert et al. (2021)
General health care	Early disease detection Personalised treatment plans Predictive analytics Surgical interventions	Mohanasundari et al. (2023)
Nurse roles and education	Administration: nursing data and documentation Nursing staffing prediction Prediction of nurse burnout Nurse education: predict student attrition Predict completion rates of undergraduates Learn to use an echocardiogram using AI‐assisted software.	O'Connor et al. (2023)

^a^ See Table 2 characteristics of literature reviews.

The nursing contexts where AI applications have been applied or have the potential for application were predominantly related to hospital care. A review of AI in clinical nursing care by Ng et al. (2022) identified six practice contexts involving documentation and patient care prediction (see Table 5). Seibert et al. (2021) described similar potential applications in general nursing care settings. Martinez‐Ortigosa et al. (2023) review flagged the potential of early disease detection and better clinical decision‐making with AI intelligence‐based support systems in nursing. Additionally, they addressed workflow optimisation and suggested that AI insights were important for nurse training and education. O'Connor et al. (2023), in reviewing the use of AI technologies in nursing, reported that AI was mainly applied in clinical practice to direct patient care, with far fewer studies addressing administration, management or education.

Several reviews examined the application of AI in clinical decision‐making and patient management, highlighting diverse applications and varying levels of impact. Jeong (2020) focused on women's health nursing in Korea, describing AI's role in predicting health‐related quality of life, medication adherence classification using support vector machines, and emergency department triage systems based on machine learning. Similarly, Chua et al. (2021) explored AI's utility in predicting delirium by analysing electronic health records. Their meta‐analysis confirmed that data mining techniques effectively predicted delirium in inpatient settings, illustrating the potential for the use of electronic health records data when combined with AI models.

In the context of emergency department triage, Sánchez‐Salmerón et al. (2022) demonstrated that random forest algorithms outperformed other triage scales in predicting patient admission or a patient's critical care needs. The study found that integrating medical records with nurses' notes enhanced prediction accuracy. In contrast, Seibert et al. (2021) highlighted the applicability of AI in long‐term care settings. AI‐enabled camera monitoring systems could detect falls among residents with dementia, reducing emergency department visits and showcasing the operational benefits of AI in nursing contexts.

Ng et al. (2022) provided a broader perspective on AI's role in nursing, identifying patient care prediction (such as falls, mortality and pressure injuries) as the most common area of research. They also noted that machine learning models improved patient monitoring efficiency across multiple studies, thus saving nurses' time and enhancing diagnostic accuracy with deep learning applications. Martinez‐Ortigosa et al. (2023) emphasised AI's potential in early disease detection, workflow optimisation, and the use of support systems for patient monitoring. Their findings indicated a 12% improvement in nurses' clinical decision‐making when ML models were implemented for disease detection.

Lastly, Choi et al. (2024) highlighted the importance of involving nurses in decision‐making processes related to AI adoption. They argued that nurses play a pivotal and critical role in ensuring AI technologies are effectively integrated into clinical workflows and patient care.

Across these literature reviews, a recurring theme emerged. While AI demonstrates substantial potential in various nursing applications, its adoption in real‐world settings requires further validation of impact through larger‐scale studies, particularly in live healthcare settings. Notably, seven AI interventions referred to in the reviews were focused on outcomes regarding clinical decision‐making.

One scoping review by Buchanan et al. (2020) stands out for its distinctive approach by analysing emerging trends and potential nursing AI applications. In the future, applications were perceived as altering patient‐centred care and nurse–patient relationships. Their key finding was to consider the use of AI technology robots and bots as an adjunct to nurse practices, aiming to reduce workloads. Emerging AI technologies described in the Buchanan review included socially assistive robots, humanoid robots, mobility robots and virtual health care assistant chatbots. It was reported that nurses are already using robots in their clinical practice for various tasks across patient populations, such as to assist with exercise sessions for older adults or rehabilitation patients; to serve as a distraction tool for pain management; to facilitate conversation and rapport; to conduct interviews or to deliver patient education. Buchanan et al.'s (2020) review provides comprehensive details about potential new applications.

Three reviews addressed the use of AI tools to classify patients' risk. In a review of delirium patient identification tools, Chua et al. (2021) questioned the quality of the included primary cohort studies to present valid evidence of the tools' benefit. Hossain et al. (2023) explored the use of natural language processing in electronic medical records to identify seven potential categories where AI applications would be beneficial, including disease classification and clinical notes analysis (see Table 2). These applications appeared promising and potentially beneficial. However, Kikuchi (2020) offered a more critical perspective on NLP in electronic medical records, suggesting that better‐structured and more precise nurse documentation would enhance the effectiveness of AI tools and support further NLP research.

### 3.4 Effective Integration of AI With Nursing Clinical Practice (RQ3)

In response to the third research question: How effectively do AI applications transfer into clinical nursing practice?, reservations were expressed in several reviews. The capacity of artificial intelligence applications in clinical nursing practice to improve patient outcomes, enhance nursing efficiency, and drive medical research has been widely reported in research outcomes. However, ethical considerations surrounding transparency, fairness and accountability remain as barriers to the direct translation of AI applications into nursing practice.

Buchanan et al. (2020) outline some risks associated with the integration of AI applications into nursing practice. These include concerns about the use of predictive analytics to identify patient risks and, hence, influence clinical decision‐making. There is a lack of transparency regarding data inputs used in algorithms and models, which makes it difficult to trace back to identify any source of error if, and when this occurs. Similarly, Mohanasundari et al. (2023) highlight drawbacks of AI, including the potential for unintended errors and bias within algorithms, and a lack of transparency in AI‐driven decisions.

Furthermore, without adequate data protection, patient privacy could be impacted by AI‐informed decisions. Although technology has the potential to improve access to care and healthcare delivery, AI may increase health service inequalities by increasing the digital divide. People in rural and remote locations may have a greater need for digital and virtual health services than those in metropolitan areas, yet lack access (Buchanan et al. 2020).

In addressing these challenges, Jeong (2020) argues that nurses need to develop a good understanding of AI principles and acquire the skills to apply AI across diverse clinical settings to ensure patient benefits. Yelne et al. (2023) suggest that more AI research, interdisciplinary collaboration, the establishment of ethical guidelines and the protection of patient rights are critical steps toward maximising AI's full potential in healthcare. As Buchanan et al. (2020) highlighted, nurses have a shared responsibility to ensure that AI‐driven changes in healthcare are implemented ethically and align with core nursing values, such as compassionate care.

## 4 Discussion

This study reports a synthesis of evidence in reviews of literature to address research questions about AI in nursing: (1) the validity and integrity of AI; (2) the areas where AI demonstrates an actual impact or potential benefit for nurses' clinical decision‐making; and (3) the extent to which AI applications transfer into nursing practice. The 16 reviews in this study identified both the benefits and challenges of AI integration in nursing.

In addressing the first research question about the validity and integrity of AI technologies in nursing, the findings underline an acute need for improved study designs and standardised reporting practices to bolster rigour and trustworthiness. The variability in research designs and reporting quality remains a concern and limits the advancement of AI in health care. For example, reviews reported limited evidence bases, often relying on primary studies with lower levels of research rigour, such as cohort or descriptive designs (Chua et al. 2021; Kikuchi 2020). Issues of heterogeneity and incomplete reporting, such as missing research details, hinder evidence synthesis and raise concerns regarding credibility, rigour and transparency (Chua et al. 2021).

Seven of the reviews employed systematic review approaches, while nine were scoping or comprehensive reviews with lower methodological rigour. No systematic reviews were based on the highest standards of evidence (systematic reviews of randomised controlled trials). Methodological deficiencies within some studies limited adherence to recommended analytical methods. For example, two reviews omitted details of their review processes, while several others failed to specify the number of primary studies included. This inconsistency reduces the overall reliability of the evidence base and may deter decision makers from implementing AI approaches.

Future research should prioritise higher‐quality study designs, particularly randomised trials, and adhere to standardised reporting frameworks. As the adoption of AI in healthcare grows, nursing research must prioritise methodological rigour and build a more credible evidence base (Dunlap and Michalowski 2024; Taylor et al. 2022).

In response to the review question asking about areas where AI demonstrates an actual or potential benefit for nurses' clinical decision‐making, responses were wide‐ranging. Some of these applications are listed in Table 5. Nursing research regarding AI technologies has demonstrated potential in predicting patient care needs, detecting early signs of disease and supporting clinical decision‐making. According to O'Connor et al. (2023), specific applications include the prediction of patient deterioration, identifying the need for critical care interventions in emergency departments, detecting patient delirium, assessing fall risks and using natural language processing of electronic health records for data mining and disease classification. Irwin et al. (2025) describe seven AI applications in use by nurses, including a symptom checker, a vital‐signs monitoring wearable, a smart medication manager and a language translation app. To adopt these applications, nurses have identified a need for education in order to integrate AI into nursing practice (O'Connor et al. 2023). They argue that nurses need specific training and continuous education.

The limited empirical validation of AI applications in real‐world nursing settings poses a problem for nurse adopters. Evidence suggests that nurses have participated in various stages of AI technology design, testing and evaluation; however, their engagement is often limited in scope or influence. von Gerich et al. (2022), drawing on Zhou et al. (2021), emphasised the need for stronger and more consistent inclusion of nurses throughout the technology research and development process to enhance clinical relevance and adoption. Furthermore, limited diversity within AI design teams remains a challenge for healthcare innovation. Ruksakulpiwat et al. (2024) emphasised that interdisciplinary collaboration, including the participation of nurses and other healthcare professionals, is essential for ensuring that AI systems align with clinical realities and patient needs. Interprofessional collaboration, including active participation by nurses, is essential for bridging the gap between AI developers and clinical users to ensure that applications are appropriately tested for real‐world relevance and effectiveness. Partnerships, for instance, between information engineers and nurses, were shown to verify the practicality of AI applications and foster innovation in nursing practice (Zhou et al. 2021). Similar to our findings, Li et al. (2022) stated that the involvement of nurses in decision‐making and during the application development and testing phases is critical to ensure that AI tools are designed with the clinical realities of nursing in mind. We concur with recommendations that nurses seek collaborations in AI research (particularly as designers) and that nurses must now make themselves known to AI designers and find a way to be included in planning decisions (Chua et al. 2021).

In exploring the practical benefits of AI for nurses' clinical decision‐making, researchers should address several key practical considerations. First, AI applications should be tested in real‐world settings to assess their functionality and effectiveness outside controlled environments. Additionally, the focus should shift to a broader exploration of the implications, benefits and challenges associated with AI in clinical nursing practice. Studies should be designed with the aim of enhancing nurses' clinical decision‐making to ensure that AI tools are aligned with nursing needs and workflows.

Finally, it is important to address a key review finding that few studies reported active use of AI applications by nurses. As echoed by Rony et al. (2024), there is a need to foster a deeper understanding among nurses of the potential of AI. Nurses must be educated on how to use AI to enhance their critical thinking and clinical decision‐making, thus equipping them with the skills necessary to incorporate these technologies into their daily practice (Irwin et al. 2025). AI literacy training, grounded in professional and ethical standards, should be integrated into nursing education at both the undergraduate and postgraduate levels, enabling nurses to possess the foundational knowledge needed to navigate and effectively utilise AI.

In response to our third enquiry regarding the translation of AI techniques into nursing practice, the evidence highlights the importance of focusing on transparency, fairness and patient data privacy. The successful integration of AI in nursing is not solely dependent on the technology itself, but also on responsible and ethical application for patient care. One of the key challenges reported in the literature is to ensure that AI applications are directly translated into nursing practice in a manner that upholds ethical standards. Challenges include ensuring the security and privacy of patient data and decision‐making where there is variability in predictors.

Furthermore, risks associated with the absence of clear ethical guidelines and policies governing the use of patient data have been raised (Yelne et al. 2023). Legal implications, particularly those related to decision‐making processes influenced by AI algorithms that may be affected by bias, also warrant careful consideration (Mohanasundari et al. 2023). An emerging issue not captured in the included reviews indicates that AI overuse can lead to user complacency regarding algorithmic bias, placing patients at risk of poor (and even incorrect) decision‐making (von der Rosenthal‐Pütten and Sach 2024).

The potential legal ramifications surrounding clinical accountability for AI adoption in nursing practice remain an area of concern. Policies addressing patients' rights to information and the protection of their data are underdeveloped. We recommend that policies addressing ethical guidelines, data privacy and legal implications of AI in nursing practice be urgently considered to ensure patient protection and the ethical use of AI.

Our findings suggest that the translation of AI into nursing practice may be improved through several key actions. First, nurses should actively voice the need for clear ethical and legal guidelines to govern the use of AI in healthcare. The development of professional and ethical principles, along with policies to guide the nursing workforce, is crucial to ensure responsible AI integration. We urge nurses to support Yasin et al.'s (2024) recommendation for greater advocacy for nurse leaders who demand a voice in AI research and policy development. Equally, it is essential for nurses to enhance their AI literacy through comprehensive education at both undergraduate and postgraduate levels (Southwick 2024). Nurses must advocate for patients' rights, including privacy, in the use of AI applications, to ensure that patient interests are central to decision‐making processes. Additionally, fostering multidisciplinary collaboration, particularly with bioethicists (Rony et al. 2024), is vital to navigate the complex ethical considerations surrounding the use of AI in nursing practice. Lastly, as echoed by Seibert and colleagues (2022), the development of relatable educational guidelines is essential to inform the nursing workforce about AI applications and empower nurses with the knowledge necessary to effectively engage with these technologies.

By implementing the recommendations outlined in this section, nurses may ensure that AI serves the best interests of patients, healthcare providers and the nursing profession. As more nurses secure a seat at the planning tables, they can contribute valuable insights on issues such as data privacy, nurse‐driven data analysis, algorithmic bias and policy development. This involvement will help ensure that AI technologies align with the core values of healthcare, benefiting both patients and healthcare providers while also supporting nurses' clinical decision‐making.

### 4.1 Limitations

As with any study, this review has strengths and limitations. Reviews of any kind will typically include studies with diverse methods and variations in the quality of reporting. This heterogeneity makes it challenging to extract and synthesise evidence. Conclusions informed by umbrella reviews are not without bias and methodological flaws, as these will have arisen in the original studies. Additionally, umbrella reviews may not capture the latest evidence if they do not include studies that have not yet been synthesised into meta‐analyses. The search terms used in this study were not exclusive; terms such as medical management, for example, might also relate to nursing practice, meaning some studies may have inadvertently been omitted. The strength of the umbrella review method is that by reporting on a greater number of studies, there can be some confidence in the aggregated synthesis and results. Additionally, however, a review of literature reviews such as this may miss reporting recent research not yet included in any review. A further consideration is that some recent reviews of AI in nursing and healthcare were excluded because they did not assist us in answering the review questions. The current review is also limited by the majority of included studies being focused on potential rather than actual applications of AI in nursing. However, a strength of our synthesis lies in its ability to identify key gaps and offer recommendations for enhancing research designs and advancing AI proficiency in nursing clinical decision‐making.

## 5 Conclusions

Artificial intelligence (AI) has the potential to support nurses' clinical decision‐making, although current evidence does not consistently demonstrate its real‐world utility in nursing. While AI has been shown to enhance prediction accuracy and assist in tasks such as nurse documentation and patient monitoring, most studies remain at the development or testing stage, with limited evaluation of their practical use by nurses. The benefits of AI for patient outcomes, cost‐efficiency and workflow remain largely theoretical due to the lack of rigorous real‐world evidence and meaningful nurse engagement.

To realise any substantive benefit from AI in nursing practice, future research must move beyond technology design and instead focus on evaluating the actual use and impact of AI tools in real‐world clinical settings. This includes designing studies that involve nurses as active participants in AI development, training and decision‐making. Only through such efforts can AI be integrated into 21st‐century nursing practice in a way that upholds professional values, enhances care quality and protects patient wellbeing.

## Author Contributions

**Robyn Cant:** conceptualisation, methodology, data curation, analysis, writing – original draft, reviewing and editing. **Colleen Ryan:** methodology, data curation, analysis, writing – original draft, reviewing and editing. **Ritesh Chugh:** writing original draft, reviewing and editing.

## Funding

The authors have nothing to report.

## Conflicts of Interest

The authors declare no conflicts of interest.

## Data Availability

The authors have nothing to report.
